# Total Usual Nutrient Intakes and Nutritional Status of United Arab Emirates Children (4 Years–12.9 Years): Findings from the Kids Nutrition and Health Survey (KNHS) 2021

**DOI:** 10.3390/nu15010234

**Published:** 2023-01-02

**Authors:** Nahla Hwalla, Lara Chehade, Lynda M. O’Neill, Samer Kharroubi, Amira Kassis, Leila Cheikh Ismail, Ayesha S. Al Dhaheri, Habiba I. Ali, Sarah Ibrahim, Fatima Al Zahraa Chokor, Maysm N. Mohamad, Wafaa Ayesh, Lara Nasreddine, Farah Naja

**Affiliations:** 1 Nutrition and Food Sciences Department, Faculty of Agriculture and Food Sciences, American University of Beirut, Riad El-Solh, Beirut P.O. Box 11-0236, Lebanon; 2 Nestlé Institute of Health Sciences, Nestlé Research Center, Société des Produits Nestlé S.A., Vers-chez-les-Blanc, 1000 Lausanne, Switzerland; 3 Whiteboard Nutrition Science, Beaconsfield, QC H9W 4L4, Canada; 4 Department of Clinical Nutrition and Dietetics, College of Health Sciences, University of Sharjah, Sharjah 27272, United Arab Emirates; 5 Department of Nutrition and Health, College of Medicine and Health Sciences, United Arab Emirates University, Al Ain 15551, United Arab Emirates; 6 Department of Public Health, College of Health Sciences, QU Health, Qatar University, Doha P.O. Box 2713, Qatar; 7 Public Health Protection Department, Dubai Health Authority, Dubai P.O. Box 4545, United Arab Emirates

**Keywords:** United Arab Emirates, children and adolescents, food intake, food consumption patterns, overweight, obesity, dietary adherence, malnutrition

## Abstract

This study aims at investigating the anthropometric status and food consumption patterns of children in the United Arab Emirates (UAE) and assessing their adherence to nutrient and dietary recommendations. It is a population-based cross-sectional survey of 690 children (4–12.9 years), from 3 major Emirates. Socio-demographic and anthropometric characteristics were collected. Dietary intakes were assessed using the 24-hour recall approach. Of the total sample, 4% were stunted, 8% were wasted and 28% were overweight/obese. A third of participating children consumed above the Estimated Energy Requirement, while the majority’s intakes carbohydrate, total fat, and protein were within the recommendations; whereas over 70% and 90% of participants consumed above the WHO daily limits of free sugars and saturated fats, respectively. Inadequate intakes of linoleic acid (36% of children), alpha-linolenic acid (91%) and dietary fiber (100%) were observed. All children failed to meet the recommendation for vitamin D and considerable proportions had inadequate intakes of vitamin A, calcium, zinc, folate, and magnesium. Compared with the American Heart Association/American Academy of Pediatrics recommendations, low dietary adherence was noted for fruits (9%), vegetables (4%), and milk/dairy (14%). These findings may be used in the development of nutritional policies aimed at improving the diets of children in the UAE.

## 1. Introduction

The healthy eating and proper nutrient intakes in childhood are fundamental for optimal growth, development, and disease prevention [1,2]. Poor dietary habits established during childhood may increase susceptibility for delayed mental and motor development that may have life-long enduring effects [3]. Accumulating evidence also suggests that poor diet among children is a strong predictor for the development of adult onset non-communicable diseases (NCDs) such as obesity, cardiovascular disease, diabetes, hypertension, and certain types of cancer [2,4,5,6,7,8]. Such diseases currently account for 71% of all deaths globally, and 74% of deaths in the Middle Eastern North African (MENA) region [9,10].

In addition to the high burden of NCDs, the MENA region harbors a double burden of malnutrition with stunting, wasting, and micronutrient deficiencies coexisting with overweight and obesity. The region has in fact been ranked second in the world for obesity prevalence rates [11]. At the same time, according to data from 2017–2019, the prevalence of undernourishment is still widespread in many countries of the region, including conflict-stricken ones such as Iraq, Libya, Somalia, Sudan, Syria and Yemen (25.2%), as well as wealthy countries such as UAE, Qatar, and Saudi Arabia (5.2%) [11]. The increasing prevalence of NCDs coupled with the double burden of malnutrition in the region may at least be partially explained by the ongoing nutrition transition with its characteristic shifts in diet and lifestyle [12].

The age groups that are amongst the most vulnerable to all forms of malnutrition are children and adolescents [13]. In fact, childhood is characterized by a period of rapid growth, which affects both macronutrient and micronutrient requirements [14]. An adequate intake of energy and nutrients is crucial to meet children’s increasing needs and lower their risk of under- or overnutrition [1,2,13]. This is particularly important in the United Arab Emirates (UAE) as thinness, overweight and obesity-associated chronic diseases have emerged as a major public health concern among children and adolescents [15,16].

The UAE is a Gulf country located in the MENA region which has flourished economically and demographically and undergone significant urbanization. Twelve percent of its population are nationals, and the remaining are expats of Arab and non-Arab origin. Eighty five percent of its national and non-national population are concentrated in the UAE’s three largest emirates (Abu Dhabi, Dubai, and Sharjah) with approximately 15% of the total population aged between 0–14 years [17]. The UAE has been experiencing a rapid socioeconomic change that may have led to shifts in dietary patterns from traditional foods rich in dairy products, vegetables, and fruits to westernized energy-dense foods rich in saturated fat, sugar-sweetened beverages, and low in fruits and vegetables amongst children [18].

Despite the growing interest and evidence framing childhood to be a time of unique nutritional needs, developmental plasticity and a critical window for optimal growth and development, limited population-based studies are available on malnutrition and dietary habits of the pediatric population in the UAE. It is in this context that this study was undertaken with the objectives of investigating the anthropometric status and food consumption patterns of children aged 4–12.9 years in the UAE and assessing their adherence to nutrient and dietary intake recommendations. The study focused on national and Arab non-national children in three major Emirates in the UAE: Abu Dhabi, Dubai, and Sharjah.

## 2. Materials and Methods

### 2.1. Study Design

This study assessed the anthropometric status, dietary intakes, and food consumption patterns of a cross-sectional sample of children living in the UAE (*n* = 690; *n* = 461 nationals; *n* = 229 non-nationals), in compliance with the Kids Nutrition and Health Study (KNHS). The KNHS study is a large-scale cross-sectional survey aimed at investigating food consumption patterns, dietary intakes and food sources of nutrients in children [19]. Data were gathered from the following Emirates: Abu Dhabi, Dubai, and Sharjah.

Stratified random cluster sampling was adopted for the recruitment of the sample. The three Emirates (Abu Dhabi, Dubai, and Sharjah) constituted the various strata. Schools, which constituted the clusters, were randomly selected. Within the schools all children meeting the inclusion criteria were invited to participate. Details about the sam-pling are described elsewhere [20].

For the UAE nationals, the calculation of sample size was based on an estimated obesity prevalence of 17% [21] with a 95% confidence interval and a 5% margin of error. The calculator of the STEP-wise Approach to Surveillance Sample Size from the World Health Organization (WHO) was utilized to perform these calculations. In addition, and for comparative purposes, a sample of Arab non-nationals was recruited at a ratio of 2:1 (national to non-national). As such the total number of participants needed was 431 nationals and 215 non-nationals. The sample of Arab non-nationals consisted of Lebanese, Jordanian, Palestinian, Syrian, Moroccan, Sudanese, Libyan, Egyptian, Algerian, Tunisian, Iraqi, Yemeni, Omani, Saudi Arabian, Bahraini, and Somalian subjects. Non-Arabs were not included in the study given the significant differences in dietary intakes, recipes, and ethnic foods between Arabs (including UAE nationals) and non-Arabs.

### 2.2. Study Population

A total of 690 children between the ages of 4 and 12.9 years were recruited from preschools and schools between June 2019 and March 2020. The study sample consisted of 461 national participants and 229 Arab non-nationals. To be eligible for participation in the study, children had to be aged between 4 and 12.9 years, without suffering from inborn errors of metabolism, chronic illness, or physical disability that could affect their eating patterns or anthropometric status. Arab non-nationals who have lived in the UAE for less than 3 years or those with mothers aged less than 18 years were excluded. Living in the UAE for a duration long enough (three years) was deemed necessary so that the children’s dietary intake reflects the country’s dietary habits.

### 2.3. Data Collection

Data collection was conducted in Arabic, by research nutritionists who had participated in an extensive training in anthropometric measurement [22,23], dietary assessment, questionnaire administration, as well as ethical conduct of research. Recruitment of participants was conducted in the preschool or school settings. Information sheets about the study were distributed to the students at school/preschool, to be shared with their parents. Only parents who agreed to take part in the study by signing the information sheet were contacted and invited to visit the school and complete the questionnaire. After explaining the study protocol and objectives and receiving written consent and assent (from children aged above 7 years), the nutritionists carried out 30 min face-to-face interviews with the caregivers at the school’s clinic, in private, to collect the data. Anthropometric measurements of participating children were obtained while a multi-component questionnaire was completed by the mothers during the interview. The questionnaire inquired about socio-economic characteristics, levels of the child’s physical activity, sleeping habits, and dietary intakes. The development of the questionnaire was based on the global KNHS protocol [19], a comprehensive review of the literature, as well as culture-specific considerations. To maintain confidentiality, data collection was performed anonymously.

### 2.4. Anthropometric Assessment

Anthropometric measurements (height and weight) were gathered for all study participants using standardized protocols [22,23] and were further evaluated using WHO AnthroPlus software [24]. Height was measured without shoes, using a stadiometer (Seca 217, Hamburg, Germany) and measurements of weight were obtained to the nearest 0.1 kg (kg) with the child wearing light clothing using a clinical balance (Seca 874, Hamburg, Germany). To ensure precision, measurements were repeated two times, and the acquired 2 values were averaged. Body Mass Index (BMI) was derived by dividing the weight (in kg) by height in meters squared [25]. The interpretation of anthropometric measurements was conducted using WHO Child Growth Criteria, which was based on the age specific z-scores. Accordingly, for children aged 4–5 years, wasting was defined based on the WHO’s BMI for Age Z-Score (BAZ) of <−2; normal status −2 ≤ BAZ ≤ +1; possible risk of overweight +1 ≤ BAZ ≤ +2; overweight +2 ≤ BAZ ≤ +3; and obese as BAZ > + 3 [26]. As for children aged 5–12.9 years, wasting was identified based on WHO’s BAZ of <−2; normal status −2 ≤ BAZ ≤ +1; overweight status +1 ≤ BAZ ≤ +2; and obese status of BAZ > +2. [27]. Stunting was identified based on Height for Age Z-Score (HAZ) of <−2.

### 2.5. Dietary Intake Assessment

Dietary intake data were collected via the 24-hour recall (24-HR) method, using the multiple pass 5-Step approach that was developed by the United States Department of Agriculture [28]. For portion size estimation, the study participants were given the choice to assess portion size in reference to standard household measures (e.g., teaspoons, tablespoons and cups), or in reference to the two-dimensional visual chart for food portions [29]. A second 24-HR was acquired from a random subsample (41%) that consisted of 284 participants, of which 70.4% were nationals and 29.6% were Arab non-nationals. This was done to estimate within-person variance when assessing usual dietary intakes.

Dietary data analysis was performed using the Nutritionist Pro Software (v 5.1.0, 2014, First Data Bank, Nutritionist Pro, Axxya Systems, San Bruno, CA, USA). Based on local recipes, traditional UAE dishes were entered into the software, as single food items. The food composition databases that were utilized for dietary intake analysis included the USDA database, and the food composition tables for the Middle East [30], in addition to nutritional information from product packaging and websites when applicable, as well as information from published studies that reported on specific UAE dishes [31,32]. Usual daily dietary intakes of energy, macronutrients and micronutrients were estimated for each child. Usual energy intakes (EI) were compared with the estimated energy requirements (EER). The latter were calculated using sex and age-specific equations that were published by the 2006 Institute of Medicine of the National Academies published Guide [33]. EER equations take into consideration four physical activity levels (PAL); sedentary (PAL 1.0–1.39), low activity (PAL 1.4–1.59), active (PAL 1.6–1.89) and very active (PAL 1.9–2.5) [34]. Nutrient intakes were also compared to the appropriate reference values such as acceptable macronutrient distribution ranges (AMDR) and dietary reference intakes (DRI) [35].

To characterize the population’s food consumption patterns, food items, as consumed, were classified into the following 10 groups: grains and grain products, fruits, vegetables, milk and milk products, meats and other protein sources, mixed dishes, savory snacks, sweets and sweetened beverages, fats and oils, and lastly, condiments and sauces. An overview of the groups and the food items within each group is shown in Supplementary Table S1.

To further assess adherence of the study sample to the dietary recommendations of the American Heart Association/American Academy of Pediatrics (AHA/AAP) [36], all recipes were broken down into their constitutive food ingredients and then categorized into the following 5 main food groups: grains, fruits, vegetables, milk and dairy, and lean meat and beans [37]. Daily intakes of these food groups were then compared with recommended number of servings by age and gender, as per the AHA/AAP.

### 2.6. Data Analysis

For analysis purposes, the sample was stratified into the following two age groups in accordance with the KNHS protocol: 4–8.9 years and 9–12.9 years.

Data analysis was performed with the Statistical Package for Social Sciences Software (v 25.0, 2013, SPSS Inc., Chicago, IL, USA ) using a significance level of *p* < 0.05. All categorical variables, such as anthropometric and sociodemographic characteristics, were analyzed using percentages and frequencies, while means ± standard errors (SE) were used to describe continuous variables. Chi-squared (χ^2^) test was selected to test for statistically significant differences among categorical variables, whereas an independent *t*-test was selected for the continuous ones.

The monthly income and the crowding index, which is defined as the total number of house occupants divided by the number of habitable rooms, were used to assess the socioeconomic status of participants. The crowding index identifies an index > 1, that is more than one person per room, as a crowded household with few economic resources [38].

The percentage of participants adhering to the food group recommendations of AHA/AAP was computed and the Chi-squared test was then used to inspect the statistical differences between age groups. Total children’s energy intakes were compared with the age and sex-specific estimated energy requirements (EER) as published by the Institute of Medicine (IOM) [33]. The usual intakes of macronutrients and micronutrients from food were estimated using the PC-side software [39,40]. To assess their adequacy, macro- and micronutrients were evaluated according to the age-specific DRIs presented by IOM [35]. Macronutrient estimated values were assessed relative to the AMDR while micronutrients were assessed according to the estimated average requirement (EAR), the tolerable upper limit intake (UL), and the adequate intake (AI). The AI values were used only in the case that no established EAR values were present.

## 3. Results

### 3.1. Household and Sociodemographic Characteristics

Table 1 displays the household and sociodemographic characteristics of the study sample. Of the total sample, 52% were females and 48% were males. Using the parents’ monthly income and the crowding index as an estimate of socioeconomic status, nationals had a significantly greater proportion of families with higher monthly income (29.1% vs. 3.5%) and a crowding index of <1 (15.6% vs. 2.2%) when compared to Arab non-nationals. In contrast, a significantly higher proportion of Arab non-national parents (65.5%) had completed higher education (university level or graduate degree) compared to nationals (39.4%).

### 3.2. Anthropometric Characteristics

**Figure 1:**
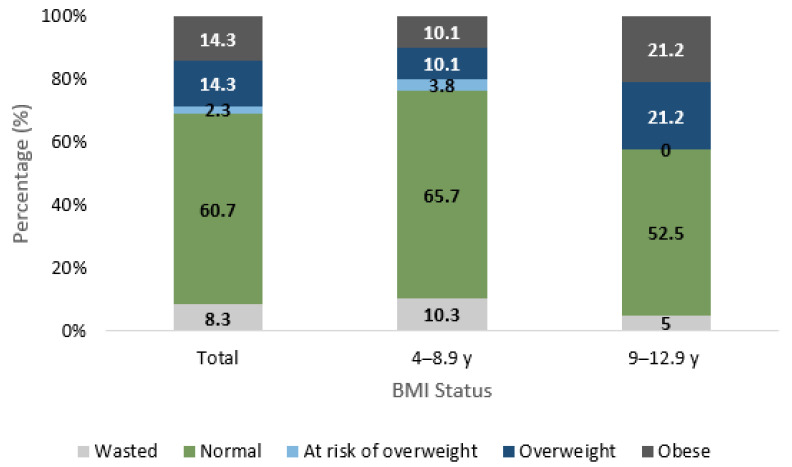
Anthropometric Characteristics of UAE Children Aged 4 to 12.9 Years Separated by Age, KNHS 2021. Significant difference in BMI status was observed between the two age groups with *p* < 0.001. At Risk of Overweight Status is only applicable to children below the age of 5 years.

The anthropometric characteristics of the study participants are shown in Table 1 by nationality and in Figure 1 by age. There were no significant differences in anthropometric status between nationals and non-nationals. However, there were significant differences by age for BMI status. For instance, the prevalence of wasting amongst 4–8.9 year-old children (10%), was twice as high compared to the older age group of 9–12.9 years (5%) (*p* < 0.001). In addition, a significantly higher proportion of children aged 9–12.9 years were obese (21%), as compared to the younger age group (10%) (*p* < 0.001). Although a higher proportion of 9–12.9 year-old children were stunted (5.0%) when compared to children aged 4–8.9 years (3.8%), these differences did not reach significance.

### 3.3. Food Consumption Patterns

Food items, as consumed by the study participants, were categorized into 10 food groups. Table 2 displays the mean intake from the various food groups, by age. Grains comprised the major source of energy for the total sample (~27%), followed by mixed dishes (~19%), and then sweets, sweetened beverages, and desserts (~17%). The contribution of fruits and vegetables to energy intake was equal or less than 5% (for each food group). Milk and milk products accounted for an average of 10% of the energy intake in the total sample. As for meats and other protein sources, this food group contributed approximately 10% of the daily energy intake in children aged 4–12.9 years. When looking at differences between age groups, older children (9–12.9 years) had a significantly greater consumption (% energy intake) of mixed dishes and savory snacks compared to younger children, and a lower intake of fruits, meats/other protein sources, sweets, sweetened beverages and desserts as well as fats and oils.

**Figure 2:**
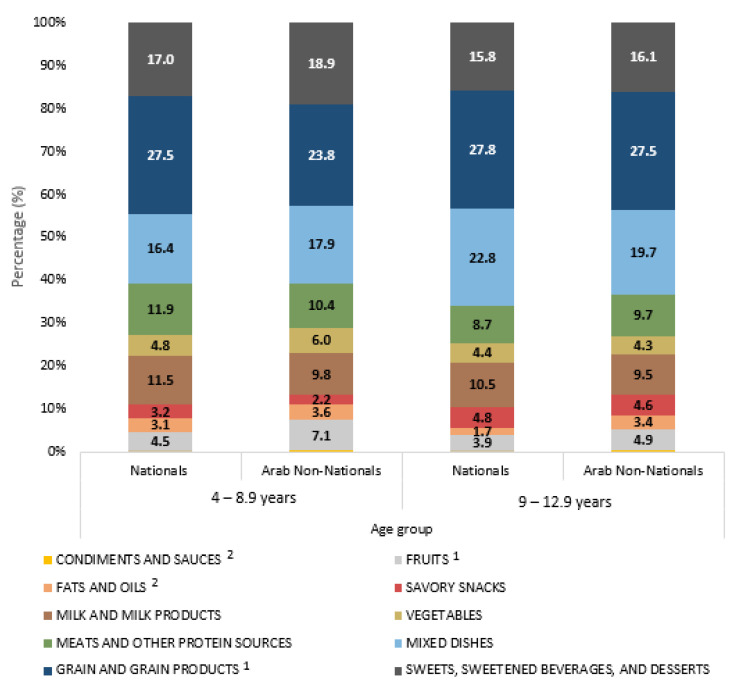
Mean percentage of energy from various food groups among UAE children aged 4 to 12.9 years separated by age and nationality, KNHS 2021. ^1^ Indicates significant difference (*p* < 0.05) between nationals and non-nationals amongst 4–8.9 year-old children. ^2^ Indicates significant difference (*p* < 0.05) between nationals and non-nationals amongst 9–12.9 year-old children.

Figure 2 displays the mean intake of the 10 food groups as a percentage of energy intake, by age group and nationality. Amongst 4–8.9 year-old children, nationals had a significantly higher consumption of grains and grain products (27.5% EI) compared to Arab non-nationals (23.8% EI), while non-nationals had a significantly higher intake of fruits (7.1% EI) compared to nationals (4.5% EI). Among the older age group, the intake of fats and oils was found to be significantly different between nationals (1.7% EI) and Arab non-nationals (3.4% EI) as well as that of condiments and sauces (0.03% EI in nationals vs. 0.27% in non-nationals).

### 3.4. Adherence to Dietary Recommendations

**Figure 3:**
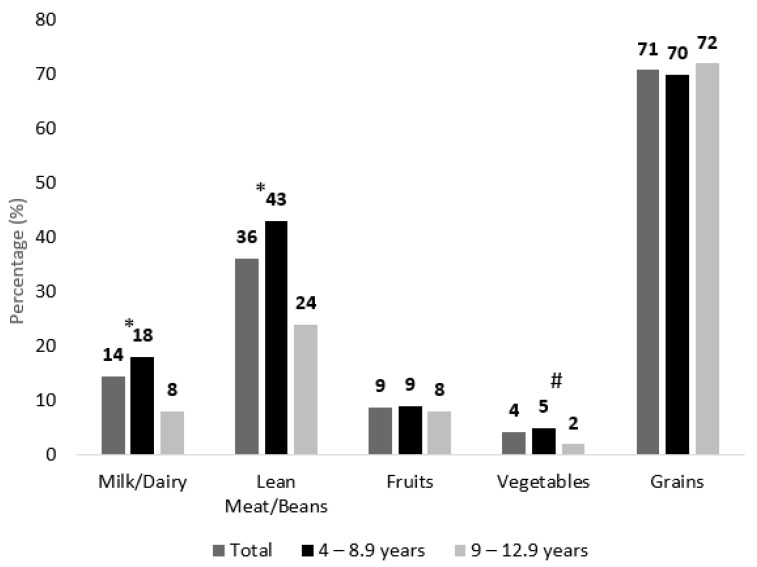
Adherence to AHA/AAP Dietary Recommendations amongst UAE Children aged 4–12.9 years (%), KNHS 2021. * Indicates significant difference (*p* < 0.05) between the 4–8.9 and the 9–12.9 years age groups. # Indicates a *p*-value of 0.052 (trend) between the 4–8.9 and the 9–12.9 years age groups.

Figure 3 displays the percentage of children adhering to the dietary recommendations of the AHA/AAP guidelines, after the disaggregation of composite/mixed dishes. Approximately a third of the children were adhering to the lean meat/bean intake recommendations (36%), with a significantly higher adherence amongst 4–8.9 year-old children (43%), compared to the older age group (24%). Adherence to milk and dairy recommendations (14% in the total sample) significantly decreased from 18% in 4–8.9 year-old children to 8% in 9–12.9 year-old children. The lowest adherence levels were noted for vegetables, with the proportions of adherence being the lowest in the older age group (2%), compared to the younger one (5%).

As for differences between nationals and Arab non-nationals, a significantly greater proportion of nationals adhered to the recommended servings of grains in comparison to non-nationals (75% vs. 63%), while a significantly lower proportion adhered to fruit recommendations (7% of nationals vs. 13% of non-nationals).

### 3.5. Energy, Macro- and Micronutrient Intakes

**Figure 4:**
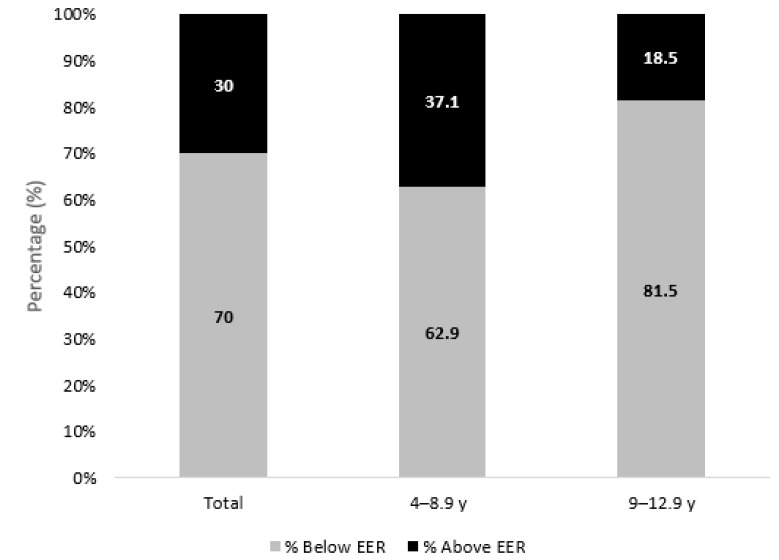
Proportions of UAE Children aged 4–12.9 years Consuming Above and Below the EER, KNHS 2021. Significant differences were observed between the two age groups with *p* < 0.001.

Energy, macronutrient, and micronutrient intakes of the study sample and their DRI compliance are presented in Figure 4 and Table 3. Approximately a third of the study sample (30%) had an energy intake above the EER, with a higher proportion in the younger group (37.1%) compared to the older one (18.5%) (*p* < 0.001). Significant differences were observed between nationals and non-nationals whereby a higher percentage of nationals had their energy intake above EER (33.2%) compared to non-nationals (23.8%) (*p* = 0.012).

Total fat consumption was within the ADMR for the majority of the sample, whereas 90% exceeded the maximum WHO recommendation for saturated fat (8% EI). In the study sample, 36.4% and 91.8% of the children were consuming below the AMDR for linoleic acid (LA) and alpha linolenic acid (ALA) intakes, respectively. For alpha-linolenic acid, the proportion of inadequacy was significantly higher amongst 4–8.9 year-old children, compared to older ones (17.7% vs. 8.1%). Although most of the participants were consuming within the AMDR for carbohydrates, none of the children aged 4–12.9 years were consuming above the AI for dietary fiber (Table 3). Free sugars intake levels exceeding the 10% EI upper limit were observed in 70% of 4–8.9 year-old and 74% of 9–12.9 years old children.

**Figure 5:**
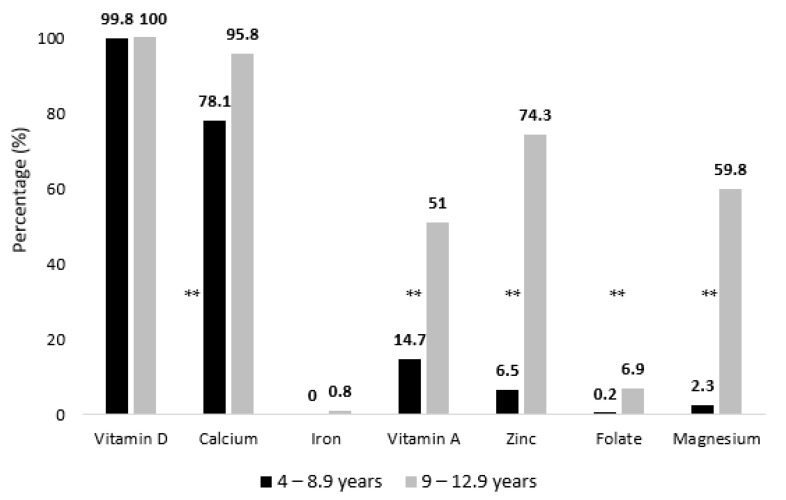
Proportions of UAE Children Aged 4–12.9 Years Consuming Below the EAR of Micronutrients, KNHS 2021. ** Indicates significant difference at *p* < 0.001.

As for micronutrient intakes, a low intake of vitamin D was evident in the study sample, whereby 100% of children aged 4–12.9 years were consuming below the EAR value (Table 3, Figure 5). Low intakes of calcium were also noted, with a higher proportion of children having inadequate intakes in the older age group (96%), compared to those aged 4–8.9 year-old (78%).

Low intakes of zinc, magnesium, and vitamin A were significantly more prevalent in the older age group (9–12.9 years) compared to younger ones, reaching as high as 74.3% for zinc, 59.8% for magnesium and 51% for vitamin A. Similarly, the proportions of subjects having low intakes of vitamin C, folate, riboflavin, vitamin B6, and vitamin B12 were significantly higher in 9–12.9 year-old children, compared to younger ones. High sodium intake was noted among 58% of children aged 4–8.9 years and 36% of children aged 9–12.9 years, while potassium intakes exceeded AI levels in only 3.5% and 3.1% of 4–8.9 and 9–12.9 year-old children, respectively (Table 3).

Among the total sample, nationals had a significantly higher proportion of children with intakes of free sugar above the WHO recommendation (74.8% vs. 65.5%, *p* = 0.010) and intakes of total fat and linoleic acid below the AMDR (11.1% vs. 6.1%, *p* = 0.037 and 41.9% vs. 25.3%, *p* < 0.001, respectively). Nationals had also higher proportions of children not meeting the AI for vitamin K (60.9% vs. 49.3%, *p* = 0.004) and not meeting the EAR for magnesium (26.5% vs. 19.2%, *p* = 0.036).

## 4. Discussion

The purpose of the study was to examine the anthropometric status and food consumption patterns of children aged 4–12.9 years in the UAE and assess their adherence to nutrient and dietary intake recommendations. The findings of this study showed that close to 29% of the study sample were overweight or obese, while 8% were wasted and 4% were stunted. A low adherence to food group recommendations was observed, particularly for fruits, vegetables and milk and dairy products. The study has also documented that the majority of 4–12.9 year-old children in the UAE had a high intake of saturated fat, and free sugar, coupled with a low intake of dietary fiber. Micronutrient inadequacies were also observed for key vitamins and minerals, and the prevalence of inadequacies was higher in the older age group (9–12.9 years) compared to younger ones (4–8.9 years).

The observed prevalence of overweight/obesity in the study sample (approximately 29%) ties well with the results on energy intake, whereby 30% of the study sample were found to exceed their estimated energy requirements (EER). The prevalence of overweight/obesity is also in line with a former study conducted on a smaller population group in the Emirate of Sharjah, where the prevalence rate was estimated at 28.2% amongst 6–11 year-old children [45]. Studies on older adolescents have reported higher estimates of overweight and obesity, such as the national Global School-based Student Health Survey that was conducted amongst children aged 13–17 years (55%) [46,47].

The higher levels of overweight and obesity noted in our study among children aged 9–12.9 years in comparison to the younger ones (4–8.9 years) agree with the results of a recent cross-sectional study performed in governmental schools in the emirate of Ras Al-Khaimah that reported on the progressive increase of obesity with age [48]. These observations may be at least partially explained by older children’s general inclination to eat meals away from home and their tendency to have more autonomy in food selection [49,50]. This is supported by a cross-sectional study conducted on a mostly national adolescent student sample in the emirate of Dubai, which showed that 67% of students reported buying food from school every day [51]. Evidence has in fact linked frequently eating out with an elevated risk of obesity, as food meals prepared away from home (including in schools) are likely to be higher in calories [52]. In addition to overweight and obesity, the study has also investigated anthropometric indicators of undernutrition. The estimated stunting prevalence within our total sample (4.2%) was found to be lower than the global prevalence of 21.3% and lower than the regional prevalence of 16.5% in children aged 6 and older [53,54]. The prevalence of wasting in our sample (8%) was also found to be lower than the global levels (11%) while being similar to that reported from other oil-rich countries (e.g., 8% in Saudi Arabia, 5% in Qatar) [55]. No significant difference was noted in the prevalence of stunting or wasting between age groups, nor between nationals and Arab non-nationals.

Unlike findings reported by a recent KNHS study conducted in Lebanon where 68% of children aged 4–13 years exceeded the AMDR for total fat [56], our results showed that only 8–11% of the children in the UAE exceeded the AMDR for this macronutrient. However, our results showed that close to 90% of children in the UAE surpassed the maximum WHO recommendation of 8% EI from saturated fat per day [43]. Similar high intakes of saturated fatty acids were previously described amongst 6–18 year-old children in the UAE as well as amongst children and adolescents in Lebanon [56,57,58]. These results highlight a public health concern given that high intakes of saturated fat have been previously linked to a higher risk of metabolic abnormalities and noncommunicable diseases in children [59,60,61]. Conversely, our study findings showed that more than a third of children in the UAE had low intakes of LA, while more than 90% had low intakes of ALA. These results are of concern as LA and ALA are both vital for children’s normal growth and development, and for proper functioning of physiological processes, such as supporting cell membrane structure, synthesizing prostaglandins, and maintaining skin health of children [62,63]. More importantly, the study results raise a concern on the adequacy of the ratio of omega 6 to omega 3 fatty acids, and its implications on the inflammatory profile in children [64,65].

Our findings also showed that more than 70% of 4–12.9 year-old children had high intakes of free sugar. In parallel, sweets, sweetened beverages, desserts were found to be a major contributor to energy intake, providing 17% of daily calories among 4–13 year-old children in the UAE. Such foods were previously shown to be the primary sources of free and added sugars across all age categories, particularly children and adolescents [2,66,67]. Similar data have been reported from other Gulf countries where approximately 90% of children consumed above the WHO limit for free sugar (10% EI) [68]. Findings stemming from clinical trials and observational studies have highlighted high sugar intake as a significant risk factor for obesity [69,70], cardiovascular disease [71,72], diabetes [73], some types of cancer [74], and non-alcoholic fatty liver disease [75,76], in addition to dental caries [77,78].

In the present study, the intakes of the different food groups were compared to the dietary recommendations set by AHA/AAP. Even though more than 2/3rd of the participating children were found to adhere to the recommended number of servings of grain and grain products, all children were found to consume below the 25 g dietary fiber recommendation set by the WHO and FAO [79]. This result concurs with previous research where inadequate dietary fiber intake was documented amongst children and adolescents across multiple countries in the MENA region [80]. This potentially indicates that the high intake of grains noted in our study is mostly in the form of refined grains, which lose valuable fiber, vitamins, and minerals in the refining process [81]. Whole grains, on the other hand, are rich in nutrients and have been reported to decrease the risk of obesity, type 2 diabetes, heart disease, and cancer, if adequately consumed [81,82,83,84,85]. Globally, roughly 3 million deaths and 82 million disability-adjusted life years were attributed to diets poor in whole-grains in 2017 [86].

Our results also showed that a high proportion of 4–12.9 -year-old children were not adhering to the recommended intakes for lean meat/beans, milk/dairy, fruits, and vegetables. Furthermore, children from the older age group (9–12.9 years) were significantly less adherent to the recommendations pertinent to lean meat/beans, milk and dairy, as well as vegetables compared to their younger counterparts (4–8-year-old children). Similar findings have been reported by a recent study conducted on the same age group in Lebanon [56]. The low proportions of children adhering to the AHA/AAP recommendations is alarming as adequate intakes of nutrient-dense food groups (such as lean meats/beans, milk and dairy, fruits and vegetables) can contribute to improving children’s nutritional status, building and maintaining healthy bones, and decreasing the risk of several chronic diseases [2]. The low consumption of these nutrient-dense foods may be explained by the remarkable economic transition that the Eastern Mediterranean Region has been undergoing in the last few decades. Such transition is accompanied by an unfavorable nutrition transition away from traditional dietary patterns high in nutrient-dense foods such as fruits, vegetables, and legumes, and more towards westernized food patterns characterized by their energy-dense and highly processed foods [18,87,88,89]. Such poor unhealthy dietary behaviors have been previously reported in the UAE. In Dubai, a cross-sectional study from 17 government schools including a mainly Emirati sample (~90%) of 1022 students aged 12–16 years reported poor dietary habits where 31% indicated drinking SSBs daily, 15% never ate fruits, 18% never drank milk, and 67% indicated buying food from school on a daily basis [51]. These behaviors, along with high intakes of sugars and saturated fats, contribute to the establishment of micronutrient deficiencies [81,90] and the risk of NCDs and mortality [91,92].

The assessment of micronutrient intakes in this study demonstrated a generally low adherence to DRIs, particularly for vitamin D, vitamin A, potassium, calcium, zinc, and magnesium. Previous research on children’s nutrient adequacy in the MENA region reported similar insufficient intakes of vitamin A, vitamin D, calcium and zinc, among school-aged children in KSA, UAE, and Lebanon [56,93]. Moreover, former studies conducted in the UAE found that over 80% of children aged 6–13 years failed to satisfy the DRI for calcium and vitamins A, D, and E [57].

The fact that all participating children did not meet the DRI for vitamin D is very concerning as this vitamin is essential for bone health and skeletal growth, with inadequate intakes contributing to rickets in young children and osteomalacia in adolescents [94]. Interestingly, despite the generous sunshine in MENA countries, the region presents several of the lowest concentrations of serum 25-hydroxyvitamin D when compared to the rest of the world [95]. Previous studies showed that the prevalence of hypovitaminosis D among children and adolescents varied between 12%–62% in several MENA countries such as Algeria, Bahrain, Egypt, Iran, Jordan, Lebanon, and Palestine [95,96]. Even higher prevalence of hypovitaminosis D (up to 99%) was reported from countries like the UAE and KSA [95]. Bone health also greatly relies on the availability of calcium, which was also inadequately consumed by the majority of children in our study (78% of 4–8.9 year-old children and 96% of 9–12.9 year-old children). The low intake of calcium is a reflection of the low intake of milk and dairy products, whereby only 14% of the study sample met the recommended number of servings from this food group. The fact that only 3% of the study sample exceeded the AI for potassium may also reflect the observed low intakes of fruits and vegetables amongst children in the UAE.

Age-based discrepancy was observed in the prevalence of micronutrient inadequacies with a higher burden in the older age group, particularly for vitamin A, zinc, calcium, vitamin C and magnesium. In fact, more than half of children aged 9–12.9 years had low intakes of vitamin A. According to the WHO, the primary preventable cause of childhood blindness in the world is vitamin A deficiency [97]. Low intakes of vitamin A can also increase the child’s vulnerability to infections, which could potentially lead to higher morbidity and mortality [97]. Zinc intake was inadequate in more than 70% of children aged 9–12.9 years. Considering its key involvement in several physiological processes, an insufficient intake of zinc may compromise physical development, immunocompetence, and reproductive and neurobehavioral functioning [98]. Intake of magnesium was also inadequate among approximately 60% of 9–12.9 year-old children. Low intakes of magnesium levels may jeopardize growth, development and energy production in children in view of the essential role it plays in bone health and development, protein synthesis, and cell regeneration and growth [99,100].

More than half of 4–8.9 year-old children and more than a third of 9–12.9 year-old children consumed above the sodium Chronic Disease Risk Reduction recommendation (1500 mg/day for 4–8.9 year-old children and 1800 mg/day for 9–12.9 year-old children) [44]. Of concern is that this high intake of sodium is accompanied by a low intake of potassium. The imbalance in the intake of these 2 minerals can contribute to pediatric hypertension, which can persist into later life, leading to heart disease and stroke [101,102]. The WHO strongly recommends the reduction of sodium intake (<2 g/day adjusted downward in accordance with caloric needs) and an increase in potassium intake (90 mmol/day adjusted downward based on the energy requirements of children relative to those of adults) for optimal blood pressure regulation in children [100,103].

This study has several strengths and limitations. One of its strengths is the fact that we have assessed usual nutrient intakes to assess dietary intakes and their adequacy amongst children. We have also measured the anthropometric characteristics of participating children using standardized protocols and procedures, rather than obtaining self-reported measures. For comparative purposes, we have recruited a sample of Arab non-nationals in addition to nationals, based on a ratio of 1:2. The results did not show significant differences between these 2 groups in anthropometric status but suggested that there may be differences in food consumption practices whereby nationals were found to have a higher consumption of grains compared to non-nationals. In contrast, Arab non-nationals were found to have a higher consumption of fruits and fats and oils. Differences in the intakes of free sugar and total fat were also observed between the groups, Thus, although the study sample is limited by its small size which does not allow for the generalizability of these differences, the results of the study highlight the need for more targeted research examining the diet and food consumption practices of the different population groups living in the UAE.

The findings of this study ought to be considered in light of the following limitations. The first potential limitation is related to the use of the 24-HR for dietary assessment, a method that may be linked with reporting bias by caregivers [104]. However, although the 24-HR approach is characterized by certain limitations such as reliance on memory and/or potential day-to-day variation in intakes, this approach was shown to give accurate estimates of energy intakes at the population level [105]. In addition, we have used the multiple pass approach to collect dietary information, which may help in reducing the limitations of the 24-HR [106]. Furthermore, to decrease interviewer errors, all of the 24-HR were administered by skilled research nutritionists who had received extensive training before engaging in data collection. It is also important to mention that all nutritionists who conducted the interviews were trained on avoiding judgmental verbal and non-verbal cues in order to minimize social desirability bias. Finally, the selected three Emirates for this study are home to 85% of the total population of the UAE, and hence the exclusion of the remaining 15% in four Emirates would represent an additional limitation.

## 5. Conclusions

In conclusion, this study documented a high intake of saturated fats and free sugars amongst school-aged children in the UAE, coupled with low intakes of alpha-linolenic acid and dietary fiber. In addition, the results highlight low adherence to the dietary recommendations for food groups, including fruits, vegetables, lean meat/beans, and milk/dairy products. Suboptimal intakes of key vitamins and minerals were also observed, including vitamin D, calcium, vitamin A, zinc, potassium, and magnesium, with the majority of these inadequacies being significantly higher in the older age group (9–12.9 years), compared to younger ones (4–8.9 years). Public health and nutrition education interventions are needed to promote and support healthier diets among children in the UAE, and hence contribute to optimal health in future generations.

## Figures and Tables

**Table 1:** Sociodemographic and Anthropometric Characteristics of UAE Children Aged 4–12.9 years, KNHS 2021.

	Total *n* (%)	Nationals *n* (%)	Arab Non-Nationals *n* (%)	*p*-Value
	690 (100)	461 (66.8)	229 (33.2)	
**Socioeconomic Characteristics**				
Age Distribution				0.077
4 to 8.9 years	429 (62.2)	276 (59.9)	153 (66.8)	
9 to 12.9 years	261 (37.8)	185 (40.1)	76 (33.2)	
**Gender**				0.029 *
Male	330 (47.8)	207 (44.9)	123 (53.7)	
Female	360 (52.2)	254 (55.1)	106 (46.3)	
**Household monthly Income ^1^**				<0.001 *
<30,000 DHS	261 (37.8)	127 (27.5)	134 (58.5)	
30,000 to 50,000 Dhs	124 (18.0)	117 (25.4)	7 (3.1)	
50,000 to 100,000 Dhs	18 (2.6)	17 (3.7)	1 (0.4)	
Don’t Know/Refused	287 (41.6)	200 (43.4)	87 (38.0)	
**Crowding Index**				<0.001 *
<1 person/room	76 (11.1)	71 (15.6)	5 (2.2)	
≥1 person/room	607 (88.9)	383 (84.4)	224 (97.8)	
**Education of Mother**				<0.001 *
Less than Elementary ^2^	11 (1.6)	7 (1.5)	4 (1.7)	
Elementary to Secondary ^3^	361 (52.3)	279 (60.5)	82 (35.8)	
University degree	294 (42.6)	160 (34.7)	134 (58.5)	
Graduate/Professional degree	24 (3.5)	15 (3.3)	9 (3.9)	
**Education of Father**				<0.001 *
Less than Elementary ^2^	5 (0.7)	5 (1.1)	0 (0.0)	
Elementary to Secondary ^3^	337 (49.3)	266 (58.2)	71 (31.4)	
University degree	297 (43.5)	157 (34.4)	140 (61.9)	
Graduate/Professional degree	44 (6.4)	29 (6.3)	15 (6.6)	
**Mother Employed**				0.187
Yes	301 (43.6)	193 (41.9)	108 (47.2)	
No	389 (56.4)	268 (58.1)	121 (52.8)	
**Father Employed**				0.124
Yes	634 (93.0)	420 (91.9)	214 (95.1)	
No	48 (7.0)	37 (8.1)	11 (4.9)	
**Anthropometric Characteristics**				
Stunting Status ^4^				0.293
Stunted	29 (4.2)	22 (4.8)	7 (3.1)	
Not Stunted	656 (95.8)	436 (95.2)	220 (96.9)	
**BMI Status ^5^**				0.892
Wasted	57 (8.3)	41 (9.0)	16 (7.0)	
Normal	416 (60.7)	279 (60.9)	137 (60.4)	
At Risk of Overweight ^6^	16 (2.3)	10 (2.2)	6 (2.6)	
Overweight	98 (14.3)	63 (13.8)	35 (15.4)	
Obese	98 (14.3)	65 (14.2)	33 (14.5)	

* Indicates significance at *p* < 0.05 (chi-square test). ^1^ US dollars = 3.67 UAE Dirhams. ^2^ Less than elementary is defined as being illiterate, not attending school, or being able to only read and write. ^3^ Elementary to secondary is defined as primary, intermediate, or high school, or technical diploma. ^4^ Stunted if HAZ <−2, not stunted if HAZ ≥−2. ^5^ Anthropometric measurements of children were in accordance with WHO classification [26,27]. ^6^ At Risk of Overweight Status is only applicable to children below the age of 5 years. Note: Some variables had missing values (Education of Father, Father Employed, Stunting, BMI Status) and therefore the percentages reported within this table are valid percentages.

**Table 2:** Mean intake of various food groups among UAE children aged 4 to 12.9 years per capita separated by age, KNHS 2021.

Food Group	Grams/Day		Kcals/Day		%Kcal/Day		*p*-Value
	4–8.9 Years (*n* = 429)	9–12.9 Years (*n* = 261)	4–8.9 Years (*n* = 429)	9–12.9 Years (*n* = 261)	4–8.9 Years (*n* = 429)	9–12.9 Years (*n* = 261)	
	Mean ± SE		Mean ± SE		Mean ± SE		
**Grain and Grain Products**	184 ± 8	210 ± 10	355 ± 12	397 ± 16	26 ± 1	28 ± 1	0.2272
**Fruits**	116 ± 8	90 ± 10	71 ± 5	55 ± 7	5 ± 0	4 ± 0	0.0055
**Vegetables**	59 ± 4	70 ± 7	69 ± 6	67 ± 8	5 ± 1	4 ± 1	0.2570
**Milk and Milk Products**	206 ± 9	209 ± 13	145 ± 6	153 ± 10	11 ± 1	10 ± 1	0.4020
**Meats and Other Protein Sources**	73 ± 4	65 ± 5	157 ± 9	128 ± 10	11 ± 1	9 ± 1	0.0093
**Mixed Dishes**	189 ± 13	256 ± 23	247 ± 16	343 ± 22	17 ± 1	22 ± 1	0.0005
**Savory Snacks**	8 ± 1	15 ± 2	42 ± 5	78 ± 12	3 ± 0	5 ± 1	0.0015
**Sweets, Sweetened Beverages, and Desserts**	237 ± 10	281 ± 16	242 ± 11	237 ± 13	18 ± 1	16 ± 1	0.1016
**Fats and Oils**	13 ± 1	9 ± 1	45 ± 3	33 ± 4	3 ± 0	2 ± 0	0.0015
**Condiments And Sauces**	2 ± 1	1 ± 0	3 ± 1	2 ± 1	0 ± 0	0 ± 0	0.2108

Note: *p*-value is for difference between age groups based on %Kcal/day from each food group.

**Table 3:** Usual Macro and Micronutrient Intake of children Aged 4 to 12.9 Years and Percent Compliance with DRIs, KNHS 2021.

Nutrients	4–8.9 Years				9–12.9 Years			
	Nutrient Intake	DRI Compliance (%)			Nutrient Intake	DRI Compliance (%)		
	Mean ± SE	>AI	<EAR/ AMDR	>UL/ AMDR	Mean ± SE	>AI	<EAR/ AMDR	>UL/ AMDR
Energy (kcal/day)	1393.3 ± 13.0	--	--	--	1465.3 ± 17.7	--	--	--
Total Fat (g/day)	48.3 ± 0.6	--	--	--	50.7 ± 0.8	--	--	--
Saturated fat (g/day)	16.4 ± 0.2	--	--	--	17.2 ± 0.3	--	--	--
Cholesterol (mg/day)	181.0 ± 2.7	--	--	--	183.4 ± 3.4	--	--	--
Total Monounsaturated fat (g/day)	16.1 ± 0.2	--	--	--	17.0 ± 0.3	--	--	--
Total Polyunsaturated fat (g/day)	9.6 ± 0.2	--	--	--	9.9 ± 0.2	--	--	--
Linoleic acid (g/day)	8.9 ± 0.2	32.9	--	--	9.3 ± 0.1	29.1	--	--
Alpha linolenic acid(g/day)	0.7 ± 0.0	17.7 **	--	--	0.8 ± 0.0	8.1 **	--	--
Docosahexaenoic acid (g/day)	0.08 ± 0.00	--	--	--	0.08 ± 0.003	--	--	--
Carbohydrate (g/day)	189.9 ± 2.0	--	0.5	--	200.0 ± 2.7	--	0.8	--
Total Sugar (g/day)	64.4 ± 0.8	--	--	--	66.1 ± 1.1	--	--	--
Free Sugars (g/day) ^1^	40.0 ± 0.6	--	--	--	43.7 ± 0.9	--	--	--
Added Sugars (g/day)	34.7 ± 0.5	--	--	--	37.3 ± 0.8	--	--	--
Protein (g/day)	52.2 ± 0.6	--	0.2 **	--	53.8 ± 0.8	--	7.3 **	--
Dietary fiber (g/day)	10.8 ± 0.1	0.0	--	--	10.8 ± 0.2	0.0	--	--
Total Fat (%)	31.3 ± 0.2	--	8.4	20.1	31.1 ± 0.3	--	11.1	19.2
Saturated fat (%) ^2^	10.6 ± 0.1	--	--	91.4	10.6 ± 0.1	--	--	88.5
Linoleic acid (%)	5.7 ± 0.1	--	36.4	0.9	5.7 ± 0.1	--	36.4	0.8
Alpha linolenic acid (%)	0.5 ± 0.0	--	90.7	0.0	0.5 ± 0.0	--	90.8	0.0
Carbohydrate (%)	54.6 ± 0.3	--	5.4	2.6	54.6 ± 0.4	--	5.0	4.6
Total Sugar (%)	18.7 ± 0.2	--	--	--	18.1 ± 0.2	--	--	--
Free Sugar (%)	11.6 ± 0.2	--	29.6	70.4	12.0 ± 0.2	--	26.0	74.0
Added Sugar (%)	10.1 ± 0.1	--	48.7	51.3	10.2 ± 0.2	--	50.6	49.4
Protein (%)	15.0 ± 0.1	--	0.0	0.0	14.8 ± 0.2	--	0.0	0.0
Vitamin C (mg/day)	71.6 ± 1.4	--	0.5 **	0.0	70.0 ± 1.8	--	11.5 **	0.0
Thiamin (mg/day)	1.2 ± 0.0	--	0.0	--	1.3 ± 0.0	--	0.8	--
Riboflavin (mg/day)	1.3 ± 0.0	--	0.0 **	--	1.4 ± 0.0	--	5.0 **	--
Niacin (mg/day)	16.9 ± 0.3	--	0.0 *	--	17.3 ± 0.3	--	1.5 *	--
Vitamin B6 (mg/day)	1.3 ± 0.0	--	0.2 **	0.0	1.3 ± 0.0	--	5.0 **	0.0
Folate (μg/d)	370.4 ± 5.0	--	0.2 **	--	388.6 ± 5.7	--	6.9 **	--
Vitamin B12 (μg/day)	3.5 ± 0.1	--	0.9 **	--	3.4 ± 0.1	--	9.6 **	--
Vitamin D (μg/day)	3.5 ± 0.1	--	99.8	0.0	3.4 ± 0.1	--	100.0	0.0
Vitamin A (RE/day)	437.9 ± 10.0	--	14.7 **	0.0	456.4 ± 12.1	--	51.0 **	0.0
Vitamin K (μg/day)	52.5 ± 1.5	42.9	--	--	58.9 ± 2.5	42.9	--	--
Calcium (mg/day)	664.0 ± 9.6	--	78.1 **	0.0	696.7 ± 13.1	--	95.8 **	0.0
Magnesium (mg/day)	188.6 ± 2.1	--	2.3 **	--	195.5 ± 2.6	--	59.8 **	--
Iron (mg/day)	10.5 ± 0.1	--	0.0	0.0	10.9 ± 0.1	--	0.8	0.0
Zinc (mg/day)	5.7 ± 0.1	--	6.5 **	0.0	5.9 ± 0.1	--	74.3 **	0.0
Sodium (mg/day) ^3^	1616.1 ±20.4	--	--	58.3 **	1718.9 ± 31.7	--	--	35.6 **
Potassium (mg/day)	1660.9 ± 16.2	3.5	--	--	1678.6 ± 22.5	3.1	--	--

AMDR: Acceptable Macronutrient Distribution Ranges, AI: Adequate Intakes, DRI: Dietary Reference Intake, EAR: Estimated Average Requirement, UL: Tolerable Upper Intake level. ^1^ Free sugars and added sugars are compared to the WHO recommendation of 10% and the AHA recommendation of 25 g, respectively [41,42]. ^2^ Saturated fatty acids compared to the UL set by WHO of 8% for children >2 years of age [43]. ^3^ Sodium intake was compared to the Chronic Disease Risk Reduction intake level of 1500 mg/day for 4–8.9 year-old children and 1800 mg/day for 9–12.9 year-old children [44]. Sources: WHO, Dietary Reference Intakes for Calcium, Phosphorous, Magnesium, Vitamin D, and Fluoride (1997); Dietary Reference Intakes for Thiamin, Riboflavin, Niacin, Vitamin B6, Folate, Vitamin B12, Pantothenic Acid, Biotin, and Choline (1998); Dietary Reference Intakes for Vitamin C, Vitamin E, Selenium, and Carotenoids (2000); and Dietary Reference Intakes for Vitamin A, Vitamin K, Arsenic, Boron, Chromium, Copper, Iodine, Iron, Manganese, Molybdenum, Nickel, Silicon, Vanadium, and Zinc (2001); Dietary Reference Intakes for Water, Potassium, Sodium, Chloride, and Sulfate (2005); and Dietary Reference Intakes for Calcium and Vitamin D (2011). * Indicates *p* < 0.05. ** Indicates *p* < 0.001.

## Data Availability

The data presented in this study are available on request from the corresponding author.

## Supplementary Materials

The following are available online at https://www.mdpi.com/article/10.3390/nu15010234/s1, Table S1: An overview of the food groups and the food items within each food group.
