# Supplementary material for: Total Usual Nutrient Intakes and Nutritional Status of United Arab Emirates Children (4 Years–12.9 Years): Findings from the Kids Nutrition and Health Survey (KNHS) 2021
Source: Nutrients. 2023 Jan 2;15(1):234. doi: 10.3390/nu15010234 (PMC9824044; doi:10.3390/nu15010234)
Supplement: Supplementary file 1 [file nutrients-15-00234-s001.zip › nutrients-2103426-supplementary.pdf]

Supplementary Material

Table S1 An overview of the food groups and the food items within each food group.

Grains and Grains Products	Whole grains, Wheat, bread, rolls, pita, saj, cereals, crackers, pretzels, kaak, pasta, rice, & other grains
Fruits	Apples, bananas, berries, citrus fruits, grapes, kiwi, melons, fresh mixed fruits, peaches, pears, pineapples, plums, pomegranate, dried fruits, 100% juices, & other fruits
Vegetables	Broccoli, greens, spinach, beets, carrots, sweet potatoes, white potatoes, cabbage, cauliflower, celery, cucumber, eggplant, green beans, lettuce, mushrooms, okra, onions, peppers, radishes, zucchini, corn, green peas, pumpkin, tomatoes/tomato sauces, & other vegetables
Milk and Milk Products	Cow's milk, cheeses, labneh 'strained yogurt', & yogurt
Meats and Other Protein Sources	Dried beans, peas, legumes, eggs, egg dishes, beef, chicken, deli meat, fish, lamb, goat, organ meats, peanut butter, raw and roasted nuts, & seeds.
Mixed Dishes	Beans and rice, other bean mixtures, beef with vegetables and/or rice/pasta, chicken or turkey with vegetables and/or rice/pasta, fish or shellfish with vegetables and/or rice/pasta, stuffed vegetables, other grain mixed dishes, pasta-mixed dishes, pizzas, rice based dishes, sandwiches, soups, & yogurt-based dishes
Savory Snacks	Corn chips, nachos, popcorn, potato chips, & puffs
Sweets and Sweetened Beverages	Candies, gelatins, Sorbets, Ice cream , frozen yogurt, pudding, milk flavorings sugar, syrups, preserves, jelly, sweet baked goods, energy drinks, fruit-flavored drinks, soft drinks, & sweetened tea & coffee
Fats and Oils	Butter, margarine, animal fats dressings, oils, & olives
Condiments and Sauces	Condiments, herbs, seasonings, gravies, & sauces
